# Artificial Intelligence in Neurosurgery: a Systematic Review Using Topic Modeling. Part I: Major Research Areas

**DOI:** 10.17691/stm2020.12.5.12

**Published:** 2020-10-28

**Authors:** G.V. Danilov, M.A. Shifrin, K.V. Kotik, T.A. Ishankulov, Yu.N. Orlov, A.S. Kulikov, A.A. Potapov

**Affiliations:** Scientific Board Secretary; N.N. Burdenko National Medical Research Center for Neurosurgery, Ministry of Health of the Russian Federation, 16, 4^th^ Tverskaya-Yamskaya St., Moscow, 125047, Russia;; Scientific Consultant, Laboratory of Biomedical Informatics and Artificial Intelligence; N.N. Burdenko National Medical Research Center for Neurosurgery, Ministry of Health of the Russian Federation, 16, 4^th^ Tverskaya-Yamskaya St., Moscow, 125047, Russia;; Physics Engineer, Laboratory of Biomedical Informatics and Artificial Intelligence; N.N. Burdenko National Medical Research Center for Neurosurgery, Ministry of Health of the Russian Federation, 16, 4^th^ Tverskaya-Yamskaya St., Moscow, 125047, Russia;; Engineer, Laboratory of Biomedical Informatics and Artificial Intelligence; N.N. Burdenko National Medical Research Center for Neurosurgery, Ministry of Health of the Russian Federation, 16, 4^th^ Tverskaya-Yamskaya St., Moscow, 125047, Russia;; Head of the Department of Computational Physics and Kinetic Equations; Keldysh Institute of Applied Mathematics, Russian Academy of Sciences, 4 Miusskaya Sq., Moscow, 125047; Staff Anesthesiologist; N.N. Burdenko National Medical Research Center for Neurosurgery, Ministry of Health of the Russian Federation, 16, 4^th^ Tverskaya-Yamskaya St., Moscow, 125047, Russia;; Professor, Academician of the Russian Academy of Sciences, Scientific Supervisor N.N. Burdenko National Medical Research Center for Neurosurgery, Ministry of Health of the Russian Federation, 16, 4^th^ Tverskaya-Yamskaya St., Moscow, 125047, Russia;

**Keywords:** neurosurgery, artificial intelligence, topic modeling in neurosurgery, natural language processing, machine learning

## Abstract

In recent years, the number of scientific publications on artificial intelligence (AI), primarily on machine learning, with respect to neurosurgery, has increased.

**The aim of the study** was to conduct a systematic literature review and identify the main areas of AI applications in neurosurgery.

**Methods.:**

Using the PubMed search engine, we found and analyzed 327 original articles published in 1996–2019. The key words specific to each topic were identified using topic modeling algorithms LDA and ARTM, which are part of the AI-based natural language processing.

**Results.:**

Five main areas of neurosurgery, in which research into AI methods are underway, have been identified: neuro-oncology, functional neurosurgery, vascular neurosurgery, spinal neurosurgery, and surgery of traumatic brain injury. Specifics of these studies are characterized.

**Conclusion.:**

The information presented in this review can be instrumental in planning new research projects in neurosurgery.

## Introduction

The concept of “artificial intelligence” is currently widely used in various fields of science and medicine. In society’s minds, the understanding of artificial intelligence (AI) is formed by the mass media, science fiction, and scientific literature. Still, often this perception does not fully reflect the reality [1]. Technically, AI is a mathematical technology that automates the solution of an intellectual problem traditionally solved by a human [2]. At the same time, the term AI denotes the section of computer science within which such solutions are developed [3]. In the latter case, AI refers to a number of mathematical and software technologies that imitate human cognitive functions to a certain extent.

The most common examples of such technologies are “computer vision” (identification of objects in images), voice and speech recognition, analysis of natural language in texts, and machine translation. In all these examples, the computer (AI) exhibits the abilities inherent to humans: i.e., seeing, listening, reading, and understanding information. However, can this imitation of human intellectual capabilities be used in medicine? The potential of these technologies lies in the automation of medical procedures, primarily in diagnosing diseases, making clinical decisions, or predicting the treatment outcome. Like machine tools automated manual labor during the industrial revolution in the 18^th^–19^th^ centuries, the computer “at the patient’s bedside” is expected to speed up clinical decisions, increase their reliability, and improve the quality and safety of medical care. The above humanistic considerations drive researchers to develop and apply AI technologies in medicine [4–7].

Methods of classical mathematical statistics, developed under conditions of data shortage, enable making scientifically based assumptions about global patterns based on a small number of observations. A large amount of data potentially hides more complex patterns — digital fingerprints of phenomena that cannot be detected without using special technologies. These technologies are being developed with the help of AI. Since the ability to process large amounts of information perceived by sensory organs is a natural function of the human brain, we intuitively call the computer programs capable of extracting and analyzing data from visual, acoustic, and tactile signals artificial intelligence.

How do AI technologies work? The mathematical apparatus of AI allows one to find and remember characteristic patterns of data that humans cannot always interpret. This process is called machine learning (ML), which is actually the mathematical search for the best solutions to systems of equations. The ML process results in a mathematical model, i.e., a function with independent variables (predictors) and parameters learned from the ML. For example, many models use the patient’s age and the severity of his/her disease as independent variables, while the output of the model is a prediction of the treatment outcome (in numeric or categorical terms). ML “learns” the parameters of such a model from a large amount of representative data.

The most promising advantages of AI technologies are their abilities to use the maximum available information (even in an unstructured form — images or text) and find complex and important patterns in it.

Neurosurgery is a field of clinical medicine that generates a large amount of data due to the routine use of high-tech medical equipment and medical information systems. These factors predispose the field of neurosurgery to the successful adaptation of AI technologies. To conceive an AI-based project in neurosurgery, it is important to understand the current demand and implementation of these technologies, as well as to identify research areas promising for using AI. This literature review aims to characterize the problems in neurosurgery that can be solved by AI technologies and to identify the areas in which these technologies are demanded.

## Methods

The literature review was carried out with PRISMA (Preferred Reporting Items for Systematic Reviews and Meta-Analyzes) guidelines and the additional use of topic modeling to objectify the selection of topics in the publications [8, 9].

The articles from journals and international conference proceedings that met the following criteria were included in the analysis:

1.   the publication was an original research article;
2.   the publication described a disease and/or treatment methods directly related to neurosurgery;
3. the paper described the use of AI technology for solving a clinical problem related to diagnosis, treatment, prognosis, rehabilitation, or prevention of a nervous system disorder;
4. neurosurgery is a potential or current field of application of the AI technology studied.

### Literature search strategy.

 Search for the literature was performed in PubMed — the US National Library of Medicine search engine (https://www.ncbi.nlm.nih.gov/pubmed/). The search query was phrased so that all documents containing the terms “neurosurgery” or “neurosurgical procedures” and terms denoting individual AI technologies (including the analysis of large data arrays and machine learning) would appear in the search results. The exact query for the PubMed search was as follows: *“neurosurgical procedures”[MeSH Terms] OR (“neurosurgical”[All Fields] AND “procedures”[All Fields]) OR “neurosurgical procedures”[All Fields] OR “Neurosurgery”[All Fields] OR “neurosurgery”[MeSH Terms]) AND (“artificial intelligence”[All Fields] OR “machine learning”[All Fields] OR “natural language processing”[All Fields] OR NLP[All Fields] OR “text mining”[All Fields] OR “fuzzy logic”[All Fields] OR “data mining”[All Fields] OR “big data”[All Fields] OR “topic model”[All Fields].*

We reviewed the search results and selected original articles that met the above inclusion criteria. The selected articles were classified by specific areas of neurosurgery; preliminary topics of these studies were worded (using professional expertise), and their number was counted.

### Topic modeling.

 The expert-provided classification of publications by topics was then objectified using topic modeling technologies: the Latent Dirichlet Allocation (LDA) algorithm and the Additive Regularization of Topic Models (ARTM) algorithm [10, 11]. Using these methods, specific sets of words characterizing each research topic (selected by experts) were identified in these article’s abstracts. By slightly varying the number of topics (a parameter of the topic model), we selected sets of words that best characterized specific topics. Finally, the results of the above two algorithms were compared, and the research topics were interpreted in accordance with the identified key words.

### Software for data acquisition and analysis.

 The primary selection and statistical analysis of the data were carried out using original software developed by the authors in the R (version 3.5.0) programming language for statistical analysis assisted with the integrated development environment RStudio. The PubMed query was performed through the programming interface (https://www.ncbi.nlm.nih.gov/books/NBK25501) from the R environment using the rentrez package (https://cran.r-project.org/web/packages/rentrez/vignettes/rentrez_tutorial.html).

We also applied the software packages XML, fulltext, tibble, dplyr, stringr, tidyr, tidytext, and topicmodels to retrieve the data on publications and analyze them in the R environment using the LDA algorithm. The ARTM model was developed using the Python programming language (version 3.6) in the Jupyter Notebook environment.

## Results

**Figure 1:**
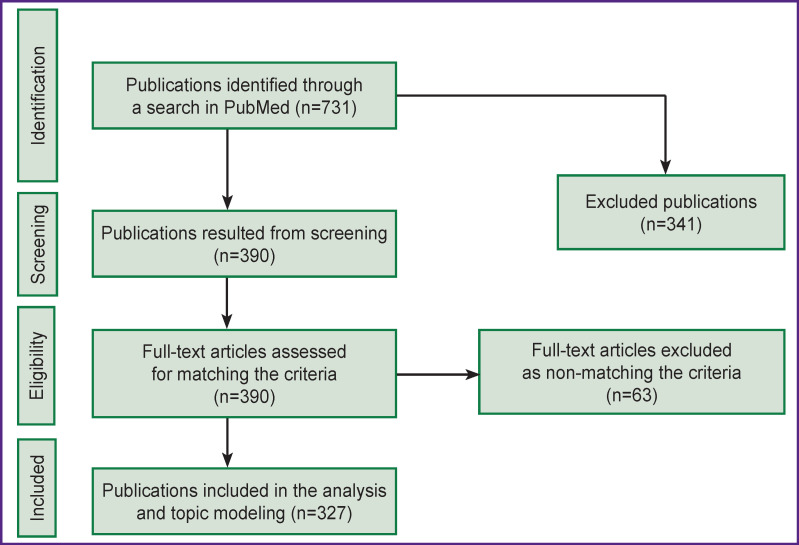
PRISMA flow diagram of the study selection process (according to PRISMA guidelines [8])

The process of selecting research articles for subsequent analysis of their topics is shown in Figure 1. The query made on July 24, 2019 in PubMed databases, produced 731 results. In accordance with the criteria for inclusion in expert analysis and topic modeling, 327 articles published from 1996 to 2019 were selected.

### General characteristics of the publications analyzed.

**Figure 2:**
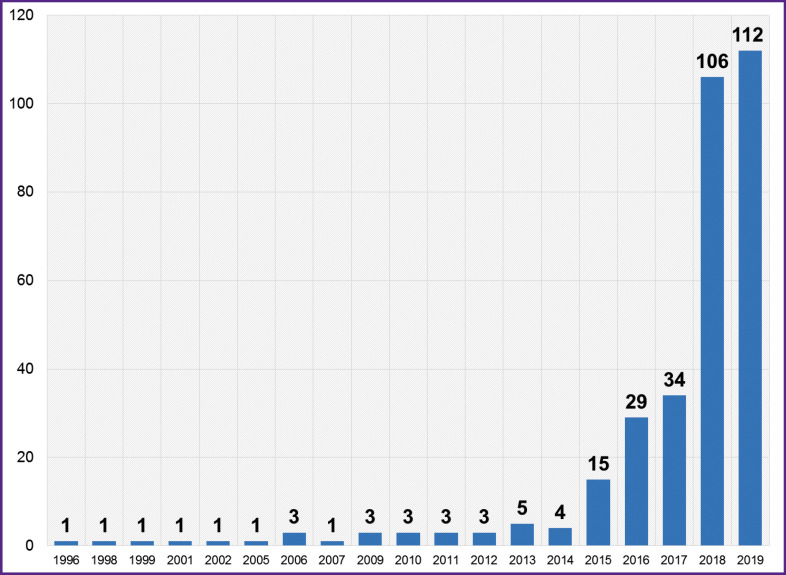
The number of analyzed studies (n=327) plotted against the year of publication

Figure 2 shows the number of selected papers arranged by the year of publication. In 2018 and the first half of 2019, there was a significant increase in the number of papers on the use of AI technologies in neurosurgery.

Most of the analyzed studies were conducted within 5 major areas of neurosurgery and one combined area:

1. neuro-oncology (n=133; 41%);
2. functional neurosurgery (n=62; 19%);
3. vascular neurosurgery (n=44; 14%);
4. spinal neurosurgery (n=29; 8%);
5. surgery for trauma brain injury (n=26; 8%);
6. other and related areas of neurosurgery (n=33; 10%).

For publications from the first 5 sections, a topic modeling analysis was carried out. The number of topics was initially selected using LDA (so that the topics differ as much as possible when interpreting the key words). Then the ARTM algorithm was applied with the same number of topics. The topics resulted from the LDA and ARTM were compared with each other. In the cases where the ARTM algorithm created two or more similar (and not separable) topics, they were assigned to the same topic under the LDA. The results of topic modeling with the LDA and ARTM algorithms (main specific key words and interpretation of topics) are presented in the Table. The complete list of 327 analyzed publications is presented in the Appendix.

**Table:** Key topics and related key words from publications in neuro-oncology, functional, vascular and spinal neurosurgery, and surgery for traumatic brain injury identified using the LDA and ARTM topic modeling algorithms

Areas of neurosurgery	Topic No.	Topic (LDA)	Key words (LDA)	Key words (ARTM)
Neuro-oncology (n=133)	1	Non-invasive tumor grading based on neuroimaging	Grade, MRI, tumor, imaging	MRI, imaging, tumor, images, grade, glioma
	2	Non-invasive molecular diagnosis based on neuroimaging	Tumor, status, imaging, mutation, gliomas, classification	Tumor, imaging, prediction, glioma, gene
	3	Segmentation of brain structures and volumetric analysis based on neuroimaging data	Segmentation, MRI, tumor, volume	—
	4	Predicting complications and treatment outcomes	Tumor, results, treatment	Results, tumor, MRI, prediction, surgery, performance, glioblastoma
				Gliomas, clinical, imaging, methods, tumor, survival, features, cancer, glioblastoma
Functional neurosurgery (including epilepsy surgery) (n=62)	1	Diagnosing epilepsy	Seizure, STN, epilepsy, stimulation, network, microelectrode	Seizure, stimulation, MRI, rate
	2	Predicting treatment outcomes in epilepsy	Seizure, outcome, classification, epilepsy, feature	Epilepsy, networks, EEG, outcome, DBS, deep, signals
	3	Detecting seizures; predicting seizures	Seizure, rate, networks, detection, neural	Surgery, seizure, stimulation, results, STN, disease, deep, network, detection, DBS
				Epilepsy, clinical, neural, lobe, TLE, EEG, DBS, seizures
	4	Diagnosing Parkinson’s disease	PD, detection	—
Vascular neurosurgery (n=44)	1	Diagnosing aneurysms; clustering aneurysms	MRA, vascular, vessel, aneurysm, segmentation, VAFA	Rupture, radiomics aneurysms, imaging
				Predicting, results, aneurysm, classification
	2	Predicting aneurysm rupture	Aneurysm, hemorrhage, prediction, biomarkers	Risk, hemorrhage, prediction, images outcome, imaging aneurysm
	3	Predicting outcomes for ruptured aneurysms	Aneurysm, rupture, unruptured, functional, score, moth	Hemorrhage, aneurysm, outcome, segmentation, prediction
				Rupture, aneurysms, predict, outcomes, stroke, consciousness
				Outcome, functional, hemorrhage, aneurysm, score, risk, outcomes, rupture
	4	Diagnosing Moyamoya disease, arteriovenous fistula, aneurysms	Aneurysm, rupture, MMD, images, DAVF, ACom	—
	5	Diagnosing intracranial carotid stenosis and predicting its complications	Stroke, results, risk, CAS, predicting, ischemic	Stroke, prediction, ischemic
	6	Predicting complications of arteriovenous malformation treatment	Predictors, radiomics, classification, epilepsy, BAVS, hemorrhage	—
	7	Predicting the outcome of treatment for arteriovenous malformation	Parenchyma, ASAH, nidal, CTP, AVM, outcome, functional	—
	8	Diagnosis and segmentation of intracranial hemorrhage	Radiomics, hematomas, prediction, physiologic	Segmentation, hemorrhage, vessel, risk, predictors, aneurysms
				Hemorrhage, cerebral, radiomics
	9	Predicting the outcome of intracranial hemorrhage	Outcome, hemorrhage, imaging, perfusion, deficit, consciousness	—
Spinal neurosurgery (n=29)	1	Non-invasive assessment of intracranial pressure	Predictive, spine, ICP	—
	2	Predicting hospital discharge options	Spinal, discharges, cord, lumbar, LSS, fusion, functional, elective	—
	3	Gait classification	Gait, images, foot, t2w, drop, results, spine, SRS, recovery	—
	4	Predicting treatment complications	Complications, surgery, risk, predicting, spine	Spine, results, prediction, predicting, complications
	5	Predicting treatment outcomes	Spinal, patients, cord, patient, surgery, models, prediction, results, spine, outcome, based, MCID, opioid, measures, predicted	Predictive, spine, spinal, quality, following, outcome, prediction, results
				Lumbar, predict, spinal, spine, LSS, results, disc, preoperative
				Spinal, spine, cord, images, lumbar, fusion
				Surgery, results, spinal, prediction, surgical, predictive, spine
Traumatic brain injury surgery (n=26)	1	Prognosis and risk of death	TBI, injury, mortality, risk	Traumatic, injury, risk, mortality, time
	2	Analysis of intracranial pressure, mean arterial pressure, cerebral perfusion pressure, and autoregulation	ICP, derived, indices, CPP, ABP, pressure	TBI, injury, traumatic, outcome, ICP, prediction, mortality
	3	Diagnosing the loss of consciousness and severity of traumatic brain injury	TBI, injury, outcome, decision	—
	4	Predicting the outcomes of traumatic brain injury	TBI, injury, outcome, traumatic, outcomes, activation	Injury, TBI, outcome, prediction, traumatic, mortality
				Injury, TBI, activation, outcomes, time

As shown in Table, the two algorithms largely lead to similar discrimination between the topics by key words. The application of AI technologies for each major area of neurosurgery will be described in more detail in the second part of this work.

## Discussion

Despite the recent development of AI technologies, machine learning, and related big data analysis methods, these technologies are yet to earn large-scale and systematic applications in neurosurgery. An objective limitation for the rapid progress in this area is, of course, the insufficient volume of high-quality data (so traditional for medicine).

Due to the increased interest in this research, the base of evidence on the performance, safety, and economic feasibility of AI methods in neurosurgery is being created. Thus, in a systematic review by Senders et al. [12], the authors described 23 studies that assessed the utility of AI technologies for solving diagnostic and prognostic tasks compared to the accuracy of medical judgments in neurosurgery. In 14 of these studies, diagnostic (classification) problems were approached using primarily neuroimaging data and electroencephalography. Seven studies addressed the methods of preoperative planning based on image analysis. In 3 studies, predicting the outcomes of neurosurgical treatment was attempted. The quoted review shows that ML can help in solving clinical problems. In 29 out of 50 (58%) tests conducted in a total of 23 studies, the results of ML were significantly better than the accuracy of conclusions made by clinical experts (p<0.05); in 18 out of 50 (36%) cases, no significant difference was found between the ML and the experts’ results; in 3 out of 50 (6%) clinical experts solved problems better than ML (p<0.05). In 4 studies assessing the performance of ML as an addition to the expert’s work, this combination was more effective than the performance of the ML or the clinician alone. This result rather confirms the hypothesis about the potential use of AI in neurosurgery.

At present, there is no enough evidence to decide on the utility, safety, and economic feasibility of AI techniques in neurosurgery; therefore, one cannot assume that AI technology is now able to replace the traditional, routine methods of medicine totally. However, given the powerful potential of AI, one can definitely suggest the need for the development and widespread testing of these methods in medical science and practice.

In the present review, methods of topic modeling are used to identify the key topics in publications. As an example of AI technologies, these methods are used to analyze texts written in a natural language. Using topic modeling, from 4 to 9 major topics (research areas) were identified for each section of neurosurgery. This approach is required for the subsequent automatic tracking of trends in a given research area and can exemplify the use of AI in biomedical science.

Below we discuss the limitations of the present work, which are important for interpreting its results. The systematic review is aimed at studying the topics and trends in using the AI solely in neurosurgery. At the same time, in related fields (for example, neuroimaging), the range of tasks solvable by AI can be much wider.

Here, we do not analyze systematic reviews on the use of AI in neurosurgery published by others since most of these papers were focused on a specific design of original studies or a specific disease and therefore had narrow inclusion criteria.

## Conclusion

To date, research into the use of AI technologies in neurosurgery has been carried out in five major areas: neuro-oncology, functional, vascular, and spinal neurosurgery, and traumatic brain injury. Using topic modeling, we identified major research topics in each of these areas. In the second part of this review, we will discuss the tasks in neurosurgery approached by AI technologies.
